# Imported malaria in Spain (2009–2016): results from the +REDIVI Collaborative Network

**DOI:** 10.1186/s12936-017-2057-8

**Published:** 2017-10-10

**Authors:** Francesca F. Norman, Ana López-Polín, Fernando Salvador, Begoña Treviño, Eva Calabuig, Diego Torrús, Antonio Soriano-Arandes, Jose-Manuel Ruíz-Giardín, Begoña Monge-Maillo, Jose-Antonio Pérez-Molina, Ana Perez-Ayala, Magdalena García, Azucena Rodríguez, María Martínez-Serrano, Miren Zubero, Rogelio López-Vélez, for the +REDIVI Study Group, Paloma Aguilera, Marta Díaz Menendez, Yolanda Meije, Joaquim Martínez-Montauti, Xavier Sanz, Isabel Pacehco Tenza, Inmaculada Gonzalez Cuello, Belén Martínez López, Mar Masiá, Sergio Padilla, Mónica Romero, José Manuel Ramos Rincón, Eduardo Malmierca, Inés Suárez-García, Juan María Herrero, Manuel Lizasoain, Pablo Rojo, Mariano Matarranz, Carlos Zarco, Jonathan Fernández Suárez, Jose Antonio Boga Ribeiro, Josune Goikoetxea Agirre, Juan Victor Sanmartín López, María Velasco Arribas, Ana Mena Ribas, María Peñaranda Vera, Israel Molina, Adrián Sánchez Montalvá, Ángel Dominguez, Nuria Serre Delcor, Diana Pou Ciruelo, Cristina Bocanegra

**Affiliations:** 1 National Referral Unit for Tropical Diseases, Infectious Diseases Department, Ramón y Cajal University Hospital, IRYCIS, Ctra Colmenar, Km 9,100, 28034 Madrid, Spain; 2 Vall d’Hebron University Hospital, Universitat Autònoma de Barcelona, PROSICS Barcelona, Barcelona, Spain; 3 Unitat Medicina Tropical i Salut Internacional Vall d’Hebron-Drassanes, PROSICS Barcelona, Barcelona, Spain; 4 La Fe de Valencia University Hospital, Valencia, Spain; 5 Alicante University Hospital, Alicante, Spain; 6 Fuenlabrada Hospital, Madrid, Spain; 7 12 de Octubre University Hospital, Madrid, Spain; 8 Valencia General University Hospital, Valencia, Spain; 9 Asturias Central University Hospital, Oviedo, Spain; 10 Albacete University Hospital, Albacete, Spain; 11 Basurto University Hospital, Bilbao, Spain

## Abstract

**Background:**

Imported malaria is a frequent diagnosis in travellers and migrants. The objective of this study was to describe the epidemiological and clinical characteristics of patients diagnosed with imported malaria within a Spanish collaborative network registering imported diseases (+REDIVI). In addition, the possible association between malaria and type of case, gender, age or area of exposure was explored.

**Methods:**

Cases of imported malaria were identified among all cases registered in the +REDIVI database during the period October 2009–October 2016. Demographic, epidemiological and clinical characteristics were analysed.

**Results:**

In total, 11,816 cases of imported infectious diseases were registered in +REDIVI’s database between October 2009 and October 2016. Immigrants seen for the first time after migration accounted for 60.2% of cases, 21.0% of patients were travellers, and 18.8% were travellers/immigrants visiting friends and relatives (VFRs). There were 850 cases of malaria (850/11,816, 7.2%). Malaria was significantly more frequent in men than in women (56.8% vs 43.2%) and in VFR-immigrants (52.6%) as compared to travellers (21.3%), immigrants (20.7%) and VFR-travellers (5.4%) (p < 0.001). Although this data was not available for most patients with malaria, only a minority (29/217, 13.4%) mentioned correct anti-malarial prophylaxis. Sub-Saharan Africa was found to be the most common region of acquisition of malaria. Most common reason for consultation after travel was a febrile syndrome although an important proportion of immigrants were asymptomatic and presented only for health screening (27.3%). Around 5% of travellers presented with severe malaria. The most prevalent species of *Plasmodium* diagnosed was *Plasmodium falciparum* (81.5%). Malaria due to *Plasmodium ovale/Plasmodium vivax* was frequent among travellers (17%) and nearly 5% of all malaria cases in immigrants were caused by *Plasmodium malariae*.

**Conclusions:**

Malaria was among the five most frequent diagnoses registered in +REDIVI’s database. Some significant differences were found in the distribution of malaria according to gender, type of case, species. Among all malaria cases, the most frequent diagnosis was *P. falciparum* infection in VFR-immigrant men.

## Background

According to the World Health Organization, between 2000 and 2015 there has been a 37% global decrease in malaria incidence and a 60% decrease in global mortality rates. However, in 2015 there were still an estimated 212 million malaria cases worldwide [1]. In the last decade, a decrease in the overall incidence of imported malaria has also been registered in most European countries although data from specialized travel networks have shown an increase in malaria cases during specific time periods [2, 3]. Specific groups appear more vulnerable, such as travellers visiting friends and relatives (VFRs), who account for the majority of imported malaria cases as documented in several studies [4, 5]. Malaria is potentially fatal especially in non-immune travellers and a favourable outcome is associated with timely diagnosis and initiation of specific correct treatment. Malaria transmission in non-endemic areas is infrequent, although isolated cases of congenital, transfusion and transplantation-associated malaria have been described [6–9]. Vector-borne transmission in non-endemic areas is unlikely but possible if, due to global climate changes, competent *Anopheles* species are introduced and become established in new areas. Imported malaria could lead to autochthonous cases if competent vectors are present in specific areas. Awareness and surveillance of imported malaria is essential for the individual patient and from a public health point of view.

The objective of this study was to describe the epidemiological and clinical characteristics of patients diagnosed with imported malaria within the +REDIVI network. The possible association between malaria and type of case, gender, age or area of exposure were explored.

## Methods

An observational prospective study included patients diagnosed with malaria and registered in the +REDIVI Collaborative Network during the period October 2009 to October 2016. The methodology used for +REDIVI has been previously published [10]. This national network includes 25 centres which share a common online database where new cases of imported infectious diseases are prospectively registered. A data collection sheet is filled out online by all members and a unique identifier code is automatically generated for each new case. Ill returned travellers/immigrants may account for more than one case in the database if a new diagnosis of an imported infectious disease is associated with a different trip. A coordinating centre is in charge of database management and quality assessment as well as ensuring proper compliance with a pre-defined protocol, which includes testing for malaria with the polymerase chain reaction (PCR) in sub-Saharan African immigrants even if asymptomatic in most centres. Ethics approval was obtained from the coordinating centre’s ethics committee and for all centres which requested it.

Variables for demographic characteristics included date of birth, gender, country of birth and main country of residence during the last 5 years. Clinical and epidemiological data included type of immunosuppression if applicable, type of traveller, date of first arrival in Spain, length of travel, date of return from travel, travel destination or country of origin for immigrants, travel risk level, pre-travel consultation attendance, indication for malaria prophylaxis, specific drug if prescribed and whether it was correctly taken. +REDIVI includes immigrants (person living in Spain but born in any other country), VFR-immigrants (immigrant travelling back from country of birth after visiting friends and relatives), VFR-travellers (person who travels back from his/her first-degree relative’s country of birth) and travellers (conventional international tourists returning from travel). In order to classify the cases, 519 diagnostic codes and 22 types of syndromes were available in +REDIVI.

A descriptive analysis was performed in order to assess gender distribution, age, type of case (immigrants, VFR-immigrants, VFR-travellers or travellers), time to presentation after travel, immunosuppression status, duration of travel, pre-travel advice, main presenting complaints and main diagnoses. Qualitative variables were expressed as relative and absolute frequencies, and quantitative data were expressed as median and 25th and 75th percentiles. Ninety-five percent confidence intervals were calculated. The Chi^2^ and Fischer exact test were used when appropriate for comparison of categorical variables. Continuous variables were compared using Student’s t test (when normally distributed) or the Mann–Whitney U test (when data were not normally distributed) and the analysis of variances (ANOVA) when several categories existed within a continuous variable.

## Results

In total, 11,816 cases of imported infectious diseases were diagnosed in +REDIVI between October-2009 and October-2016. Of these, 6289 (53.2%) cases occurred in women. The database included 2479 travellers (21.0%), 218 VFR-travellers (1.9%), 2001 VFR-immigrants (16.9%), and 7118 immigrants (60.2%). Most prevalent diagnoses in +REDIVI were Chagas disease (27.1%), eosinophilia (17.4%), strongyloidiasis (9.2%), latent tuberculosis (8.5%) and malaria (7.2%). For all other diagnoses, prevalence was below 5%.

In total, 850 cases of malaria (7.2%) were identified among the 11,816 registers. Malaria cases were significantly higher (p < 0.001) among men (56.8%) than among women (43.2%). Median age was 35.6 (27.9–44.0) years and there were 45 (5.3%) immunosuppressed patients among malaria cases. Malaria cases were not associated with age or immunosuppression status. Malaria cases were especially prevalent among VFR-immigrants as compared to other types of cases: more than 50% of malaria infections were in VFR-immigrants (p < 0.001). As for duration of travel, this was significantly longer in travellers than in VFRs (p < 0.001). Finally, median elapsed time between arrival and first consultation was higher for immigrants than for other types of patients (p < 0.001) (Table 1).

**Table 1:** Main characteristics of malaria cases

	MALARIA		*p*
	n = 850		
Women (n, %)	367	43.2	< 0.001
Age (median, P_25_–P_75_)	35.6	27.9–44.0	0.57
Immunosuppression (n, %)	45	5.3	0.52
Type of case (n, %)			< 0.001
Immigrants	176	20.7	
VFR-immigrants	447	52.6	
VFR-travellers	46	5.4	
Travellers	185	21.3	
Travel duration in days (median, P_25_–P_75_)			< 0.001
Immigrants	NA		
VFR-immigrants	30	24–61.3	
VFR-travellers	48	30–120	
Travellers	60	25–240	
Elapsed time between arrival and consultation in days (median, P_25_–P_75_)			< 0.001
Immigrants	16.5	7–77	
VFR-immigrants	11	6–20	
VFR-travellers	13	5.8–20.5	
Travellers	10	4–27	

*p* Chi^2^ *p* value for categorical variables, Student’s *T* test *p* value for continuous variables and analysis of variance (ANOVA) for continuous variables with several categories, *NA* not applicable

As for regions of acquisition of malaria, cases were mainly acquired in sub-Saharan Africa. However, among travellers, a proportion of cases were associated with travel to Latin America and Asia (5.0% for both). The most prevalent species of malaria was *P. falciparum* in the four groups (693/850, 81.5%). If species and geographic distribution are considered, the most frequent diagnosis was *Plasmodium falciparum* diagnosed in VFR-immigrants from sub-Saharan Africa. Malaria due to *Plasmodium ovale/Plasmodium vivax*, was frequent among travellers (17%) and nearly 5% of all malaria cases in immigrants were caused by *Plasmodium malariae* acquired in sub-Saharan Africa. Severe malaria cases were especially prevalent in travellers (5.5%) (p = 0.01). No fatal cases were recorded (Table 2).

**Table 2:** Geographical distribution by type of case and distribution of malaria species and severe malaria by type of case

	Immigrants		VFR-immigrants		VFR-travellers		Travellers	
	N = 176		N = 447		N = 46		N = 181	
	n	%	n	%	n	%	n	%
Subsaharan Africa	168	95.5	433	96.9	45	97.8	163	90.0
*P. falciparum*	140	83.3	380	87.8	39	86.7	123	75.5
*P. malariae*	8	4.8	5	1.2	1	2.2	1	0.6
*P. ovale*	4	2.4	7	1.6	2	4.4	14	8.6
*P. vivax*	2	1.2	0	0.0	0	0.0	7	4.3
Unknown	10	6.0	29	6.7	1	2.2	14	8.6
Mixed infection	4	2.4	12	2.8	2	4.4	4	2.5
Central and South America	2	1.1	9	2.0	0	0.0	9	5.0
*P. falciparum*	0	0.0	3	33.3	0	0.0	4	44.4
*P. ovale*	0	0.0	0	0.0	0	0.0	1	11.1
*P. vivax*	2	100.0	5	55.6	0	0.0	3	33.3
Unknown	0	0.0	1	11.1	0	0.0	1	11.1
Asia	6	3.4	5	1.1	1	2.2	9	5.0
*P. falciparum*	0	0.0	2	40.0	0	0.0	2	22.2
*P. vivax*	5	83.3	3	60.0	1	100.0	6	66.7
Unknown	1	16.7	0	0.0	0	0.0	1	11.1
Severe malaria	2	1.1	6	1.3	1	2.2	10	5.5

No cases were acquired in Europe, Australasia-Oceania, North Africa or North America

Information on whether anti-malarial prophylaxis had been taken correctly was only available for 1050 cases in the database. For patients with malaria, this information was recorded for only 217 (25.5%) out of the 850 cases and 29 out of 217 (13.4%) travellers/VFR travellers/VFR immigrants referred having taken the medication correctly. As expected, the percentage of patients referring correct anti-malarial prophylaxis was significantly (p < 0.001) lower among malaria cases (13.4%) than among non-malaria cases (46.1%).

Considering only the subgroup of travellers with *P. ovale*/*P. vivax* malaria, these patients had taken malaria chemoprophylaxis correctly significantly more frequently than those with non-*P. ovale*/*P. vivax* malaria (35.5%, 11/31 vs 2.7%, 4/150, p < 0.001). Among travellers with *P. ovale*/*P. vivax* malaria who had taken correct prophylaxis, 45.5% (5/11) had taken atovaquone–proguanil and 27.3% had taken mefloquine (3/11). Travellers with *P. ovale*/*P. vivax* malaria presented a median of 47 days (9–121) after travel whereas travellers with non-*P. ovale*/*P. vivax* malaria presented after a median of 9 days (3.75–21), although this difference was not statistically significant (p = 0.79).

With regards to reasons for medical consultation after travel, most patients with malaria (79.8%) presented with only one syndrome regardless of the type of patient (p = 0.79). Although the most frequent reason for consultation for the four groups was a febrile syndrome, a substantial percentage of immigrants (48/176, 27.3%) presented with no symptoms, for health screening only. Of note, 9/447 VFR-immigrants (2%) and one traveller (originally from Burkina Faso but travelled to Haiti) were also asymptomatic at presentation.

## Discussion

Imported malaria may still be considered a significant entity in non-endemic countries. A recent review highlighted this problem especially when considering the current international goal for global malaria eradication: the meta-analysis of imported malaria in the last decade covering more than 50,000 individual cases identified France and the UK as the countries receiving the highest number of cases, with over 4000 reported cases yearly on average [11]. This current series underscores this issue as over 120 yearly cases on average were registered within this national network and imported malaria was among the top five established diagnoses. According to WHO data, there was an average of 500 imported cases of malaria per year in Spain between the years 2009 and 2015 [12]. Even accounting for possible under-reporting at the national level of official epidemiological surveillance data for communicable diseases, the +REDIVI Collaborative Network provides additional data on a significant proportion of all imported malaria cases in Spain. This is an established network in Spain and although not all centres attending imported infections are included, data on over 11,000 patients were available for analysis. With respect to other similar series such as the Geosentinel network, a higher proportion of malaria cases were diagnosed in +REDIVI (7.2% of all diagnoses compared with 2% in the Geosentinel series) although this may be due to the fact that both symptomatic and asymptomatic patients were included and there was a greater representation of immigrants (both VFR-immigrants and conventional immigrants) in the current analysis (VFRs accounted for 15% of travellers in the Geosentinel series) [13].

VFRs are a high risk group for malaria. This has been previously documented in other series where these travellers account for more than 70% of malaria cases in some studies [4, 5]. In this study, malaria was significantly more frequent in VFR-immigrants with more than 50% of malaria infections diagnosed in this group, even though the median duration of travel was shorter than for travellers, probably reflecting the higher risk associated with VFR travel.

Pre-travel advice and preventive measures should therefore be especially directed to this group. Interestingly, no cases of malaria were seen in short-term travellers (< 25 days) and occurred mainly in medium-long term travellers in this group (probably higher risk subgroups of travellers such as aid workers and expatriates). Of concern, malaria chemoprophylaxis continues to be poorly adhered to. A longer elapsed time from arrival to consultation was found for immigrants probably reflecting a degree of semi-immunity (some of these patients were asymptomatic). The most frequently diagnosed case was in VFR- immigrants with *P. falciparum* imported from sub-Saharan Africa and this finding is similar to that found in other series identifying these patients as high risk [4]. An important proportion of non-falciparum imported malaria was also found in the study (9%, not including mixed infections and infections where species was not determined), mainly due to *P. ovale*/*P. vivax* malaria in travellers. Although this data was not available for most patients with malaria, only a minority (13.4 %) mentioned correct anti-malarial prophylaxis. Of note, travellers with *P. ovale*/*P. vivax* malaria presented later than those with non-*P. ovale*/*P. vivax* malaria and in some cases despite referring “correct” anti-malarial prophylaxis (due to a lack of a causal effect of the drugs used). This should alert clinicians to the possibility of infections which may not be easily diagnosed due to later presentation even with correct chemoprophylaxis use [14, 15]. In addition, these infections may be missed if only rapid diagnostic tests are used [16].

As expected, the most frequent presenting symptom for all groups was fever, underscoring the importance of recognizing the possibility of malaria in any person returning from an endemic area with fever in order to minimize diagnostic delay. According to ECDC surveillance data for 2014, approximately 5200 cases of imported malaria occur in EU countries annually [17], and around 1/3 of falciparum malaria cases may progress to severe malaria [18]. In this series around 5% of travellers presented with severe malaria, but this figure was lower (19/850, 2.2%) if all groups are considered probably reflecting the large proportion of immigrants in the study who may have a degree of semi-immunity. In fact, over 5% of malaria cases were diagnosed in asymptomatic immigrants, as nucleic acid testing by PCR has proven a sensitive method for screening, detecting infections with low parasite densities which are not infrequent in this group [19]. Detection of submicroscopic infections is relevant as low-grade asymptomatic parasitaemias have been described to persist for over 24 months in recently arrived immigrants. These persons may infect competent vectors in non-endemic areas acting as unidentified reservoirs and contributing to transmission in areas where malaria has been eliminated [20]. *Anopheles atroparvus*, a competent vector for Asian strains of *P. vivax*, is widely distributed in Spain. A recent publication has reported an autochthonous case of introduced malaria caused by *P. vivax* in Spain in 2014, where the strain was identical to that of another imported case from Pakistan [21]. In 2010, another autochthonous case of *P. vivax* malaria was diagnosed in a patient with no history of travel to endemic areas. The patient lived in an area where the vector *An. atroparvus* is present but the source of the infection could not be identified [22]. Local transmission of malaria should be very infrequent as the number of yearly imported *P. vivax* cases in Spain is low (an estimated < 50/year) and *An. atroparvus* is refractory to African strains of *P. falciparum* [21]. However, *P. vivax* gametocytes develop early in the course of infection and this species can produce liver hypnozoites causing relapses and, therefore, the risk of vector-borne transmission exists. In addition, if other competent *Anopheles* species are introduced due to climate change and become established in non-endemic areas the risk of local transmission may be increased. Although rare, congenital transmission or transmission by blood transfusion or organ transplantation from an infected donor could also occur years after leaving malaria-endemic areas.

*Plasmodium falciparum* was the most frequently diagnosed species in the series. This is in accordance with global prevalence of this species and also with other reports of imported malaria where *P. falciparum* is the most commonly reported species among imported cases of malaria, mainly in patients returning from sub-Saharan Africa [2]. Severe malaria cases occurred mainly in travellers highlighting the need for early diagnosis in non-immune patients.

Limitations of the study were that the data was collected mainly at specialised travel clinics and all cases of imported malaria nationally were not included, thus precluding a more complete overview of imported malaria in Spain during the study period. Also, the network recorded the main clinical and epidemiological data but more detailed information such as specific laboratory parameters for particular cases was not included and some data, such as chemoprophylaxis use, was not available for all patients.

## Conclusions

Malaria is a frequent diagnosis in returning travellers. Significant differences may be found in the distribution of malaria according to gender, type of case and species. Among all malaria cases, the most frequent diagnosis was *P. falciparum* infection in VFR-immigrants acquired in sub-Saharan Africa. Immigrants, VFR-travellers and travellers with malaria also presented mainly with *P. falciparum* infections from sub-Saharan Africa. However, *P. ovale* and *P. vivax* infections were also prevalent among travellers. This highlights the need for specialized, individually patient-tailored pre-travel advice.
